# Knowledge, Attitudes, and Practices Regarding Periodontal Diseases Among First-Year Undergraduates of Different Faculties of Krishna Vishwa Vidyapeeth University

**DOI:** 10.7759/cureus.67042

**Published:** 2024-08-16

**Authors:** Bhavana J Jadhav, Siddhartha Varma, Girish Suragimath, Sameer A Zope, Apurva V Kale

**Affiliations:** 1 Periodontology, School of Dental Sciences, Krishna Vishwa Vidyapeeth (Deemed to be University), Karad, IND

**Keywords:** physiotherapy, pharmacy, periodontal disease, nursing, medical, first-year undergraduates, dental

## Abstract

Introduction

Periodontal diseases, encompassing gingivitis and periodontitis, are prevalent and complex conditions with significant implications for overall health, particularly in India. This study aimed to assess the knowledge, attitudes, and practices (KAP) regarding periodontal diseases among first-year undergraduate students across various faculties at Krishna Vishwa Vidyapeeth Deemed to be University, Karad.

Methods

A cross-sectional questionnaire-based survey was conducted among 200 students from the medical, dental, physiotherapy, pharmacy, and nursing faculties. Descriptive statistics and Pearson's chi-squared test were utilized for data analysis.

Results

They revealed suboptimal oral hygiene practices among participants, with only 30% reporting twice-daily tooth cleaning. Additionally, less than half of the participants used soft toothbrushes and fluoridated toothpaste. Notably, 40% had never visited a dentist. While 86% recognized the correlation between gum and systemic health, significant proportions had experienced symptoms of gum disease. Dental first-year undergraduate students have a better understanding, attitude, and practices toward periodontal diseases, while nursing first-year undergraduate students displayed the lowest KAP scores compared to other faculties.

Conclusion

The first-year dental students demonstrated a better understanding, attitude, and behavior towards periodontal diseases compared to students in other faculties. Our study emphasizes the necessity of incorporating oral and periodontal health education into both healthcare professional curricula and those of higher secondary school students. Such educational programs will empower individuals to enhance their oral and systemic health. Moreover, equipping medical and paramedical practitioners with proper knowledge of periodontal health will ultimately lead to improved oral health outcomes for the general population.

## Introduction

Periodontal diseases, including gingivitis and periodontitis, are among the most common diseases. Chronic periodontitis is an infectious inflammatory disease caused by the bacteria of the dental plaque, resulting in the progressive destruction of the tissues that support the teeth, i.e., the gingival, the periodontal ligament, cementum, and the alveolar bone [1]. The increase in the prevalence of periodontal disease can be attributed to various factors, including poor oral health awareness [2]. Maintaining periodontal health is crucial for overall health. Several biological pathways may connect periodontal infections with systemic diseases. These pathways include (a) oral-hematogenous spread of periodontal pathogens and their direct effects on target organs, (b) transtracheal spread of periodontal pathogens and their direct effects on target organs, and (c) oral-hematogenous spread of cytokines and antibodies with effects on distant organs [3]. Proper oral hygiene, including tooth brushing, flossing, and regular dental visits, can prevent oral diseases [4]. Evidence of an interdependent relationship between periodontal health and systemic diseases allows healthcare professionals to identify such diseases [5]. The knowledge and oral hygiene behaviors of medical and paramedical students significantly impact oral health education for individuals and groups and serve as role models for their patients and the broader community [6]. Therefore, this study evaluated the knowledge, attitude, and practices related to periodontal diseases among first-year undergraduates in various faculties of Krishna Vishwa Vidyapeeth Deemed to be University, Karad.

## Materials and methods

Study design

This cross-sectional study was conducted among 200 first-year undergraduate students enrolled in various health science faculties, including medical, dental, physiotherapy, pharmacy, and nursing, at Krishna Vishwa Vidyapeeth University in Karad, Maharashtra, India. Before beginning the study, we obtained ethical clearance from the ethics review committee of Krishna Vishwa Vidyapeeth, with the ethical clearance protocol number 200/2022-2023. The participant information sheet was provided, and electronic consent was obtained from all the participants before enrolling in the study. The study randomly included forty students from each facility. To avoid contributor bias and information bias, all the participants who had joined within the first month from the commencement of their respective courses were enrolled in the study. Anonymous responses were collected to minimize bias and encourage more honest participant feedback. Incompletely filled-out questionnaires were excluded from the study.

Sample size

The minimum calculated sample size was 196, so a rounded sample size of 200 was obtained based on the level of significance (alpha error of 5% and power of 80%) using the formula (n=(Z1)2 (P(1-P))/d2).

Pre-validation and pre-testing of questionnaire

The questionnaire was pre-tested and pre-validated by protocol committee experts, including subject experts visiting from other universities. A pilot study among 30 participants was also conducted to assess the reliability and validity of the questionnaire. A specially designed open-ended Google form questionnaire was created, consisting of 20 questions, focusing on three domains: knowledge, attitude, and practices.

Distribution of questionnaire

The email addresses or WhatsApp numbers of first-year undergraduate students were collected by contacting the five different faculties of Krishna Vishwa Vidyapeeth University. After that, the digital consent form and questionnaire link were emailed to the students enrolled in the study. Only responses from the participants who filled out both the consent form and questionnaire were considered for statistical analysis.

Data collection

Data were gathered from 200 first-year undergraduates, with 40 students from each of the five faculties: medicine, dentistry, pharmacy, physiotherapy, and nursing.

Statistical analysis

Descriptive analysis involves presenting the explanatory and outcome variables in terms of frequency and proportions for categorical variables. In this study, Pearson's chi-squared test was used to compare the categorical responses among different faculties. All statistical analyses were performed using the IBM Statistical Package for the Social Sciences (SPSS) statistical software (SPSS version 25; IBM, Armonk, NY, USA). The p-value <0.05 was considered significant.

## Results

A total of 200 first-year undergraduates from five different faculties of Krishna Vishwa Vidyapeeth participated in the study. They were given a questionnaire with 20 questions focusing on knowledge, attitude, and practices related to dental hygiene. The findings showed that only 60 participants (30%) reported cleaning their teeth twice daily, even though brushing was the most commonly used method for cleaning teeth. Additionally, less than 100 participants (50%) used soft toothbrushes and toothpaste for cleaning their teeth. The study also revealed that 106 participants (53%) brushed their teeth for one to three minutes and that nearly half of the participants (48.5%) changed their toothbrushes once every three months. In terms of brushing techniques, 84 individuals (42%) used a circular motion, while 54 (27%) used a random motion for brushing. When it comes to oral hygiene products, 85 participants (42.5%) used mouthwash, and only 10 participants (5%) used dental floss. Additionally, 26 participants (13%) used both mouthwash and dental floss for cleaning. The study found that a total of 171 participants (85.5%) cleaned their tongues daily, with 87 (43.5%) using toothbrushes and 77 (38.5%) using tongue cleaners. Furthermore, almost half of the participants used fluoridated toothpaste. Regarding dental visits, 80 participants (40%) had never visited a dentist before (Table 1).

**Table 1:** Level of knowledge, attitude, and practices of participants (questions 1-10). *p-value <0.05: Level of statistical significance.

S. no	Variables	Responses	Percentage	Number	p-value
1	How many times do you clean your teeth?	Once a day	24.5%	49	0.000*
		Twice a day	30%	60	
		More than twice a day regularly	0%	00	
		More than twice a day irregularly	2.5%	05	
2	What kind of brush do you use?	Hard	2%	04	0.006*
		Medium	43.5%	87	
		Soft	47%	94	
		Ultrasoft	7.5%	15	
3	Time you spend on brushing	1-3 min	53.0%	106	0.003*
		3-5 min	38.0%	76	
		More than 5 min	6.0%	12	
		Not sure	3.0%	06	
4	How often do you change your toothbrush?	Once in month	25%	50	0.041*
		Once in three months	48.5%	97	
		Once in six months	21.0%	42	
		Every year	5.5%	11	
5	How do you brush your teeth?	Horizontal motion	19.5%	39	0.283
		Vertical motion	11.5%	23	
		Circular motion	42.0%	84	
		Randomly	27.0%	54	
6	Any other oral hygiene aids you use with brushing?	Mouthwash	42.5%	85	0.111
		Dental floss	5.0%	10	
		Both	13.0%	26	
		None	39.5%	79	
7	Do you use fluoridated toothpaste?	Yes	49.5%	99	0.000*
		No	19.5%	39	
		Not sure	31.0%	62	
8	Do you clean your tongue regularly?	Yes	85.5%	171	0.158
		No	7.5%	15	
		Not sure	7.0%	14	
9	If your response for the above question is "Yes," then which dental aid you use for cleaning your tongue?	Tongue cleaner	38.5%	77	0.018*
		Toothbrush	43.5%	87	
		Tongue brush	6.5%	13	
		Others	3.0%	6	
10	When was your last dental visit?	Three months ago	36.5%	73	0.138
		Six months ago	9.0%	18	
		One year ago	14.5%	29	
		Not visited yet	40%	80	

While evaluating the knowledge of first-year undergraduates from all five professionals about the relationship between systemic diseases and periodontal health, 172 participants (86%) knew about this relationship. While evaluating the self-perception results for bleeding gums, loose teeth, and pus discharge 102 participants (51%) did not experience any of these symptoms of periodontal diseases, while 137 (68.5%) were aware that gum disease is one of the reasons for bad breath. It was found that 195 (97.5%) of the participants believed that chewing tobacco has negative impacts on gum health. Out of all the participants, 81 (40.5%) had never undergone teeth cleaning. Almost half of the participants, 97 (48.5%), did not provide a reason for not cleaning their teeth, while 52 (26%) claimed that lack of time was a factor. More than half of the participants experienced sensitivity, but only 79 (39.5%) of them used desensitizing toothpaste. Additionally, 142 (71%) of the participants were aware of the periodontology specialty (Table 2).

**Table 2:** Level of knowledge, attitude, and practices of participants (questions 11-20). *p-value <0.05: Level of statistical significance.

S. no	Variables	Responses	Percentage	Number	p-value
11	Do you think that there is any correlation between gum health and systemic health?	Yes	86.0%	172	0.000*
		No	4.5%	9	
		Not sure	9.5%	19	
12	Have you ever experienced the symptoms of gum disease like bleeding gums, loose teeth, bad breath, and food lodgement?	Yes	42.5%	85	0.025*
		No	51.0%	102	
		Not sure	6.5%	13	
13	Do you know that gum disease is one of the main reasons for bad breath?	Yes	68.5%	137	0.379
		No	17.0%	34	
		Not sure	14.5%	29	
14	Do you think the habit of tobacco chewing has any effect on gum health?	Yes	97.5%	195	0.126
		No	1.0%	2	
		Not sure	1.5%	3	
15	When was the last time you had undergone cleaning of your teeth?	Three months ago	37.0%	74	0.520
		Six months ago	5.5%	11	
		One year ago	17.0%	34	
		Never done	40.5%	81	
16	If your response to the above question is Never done, what is stopping you from getting your teeth cleaned?	Fear of pain	11.0%	22	0.041*
		Lack of time	26.0%	52	
		Fear of side effects	7.5%	15	
		Prolonged treatment plan	7.0%	14	
		Not responded	48.5%	97	
17	Have you ever experienced sensitivity?	Yes	60.5%	121	0.003*
		No	29.0%	58	
		Not sure	7.5%	15	
		Not responded	3.0%	6	
18	If your response for the above question is "Yes," then what treatment have you undergone for sensitivity?	Used desensitizing toothpaste	39.5%	79	0.191
		Tooth filling	14.0%	28	
		Root canal treatment	10.5%	21	
		Fluoride application	0.5%	1	
		Not responded	35.5%	71	0.116
19	Have you ever experienced bleeding gums while brushing your teeth?	Yes	40.5%	81	
		No	55.0%	110	
		Not sure	1.5%	3	
		Not responded	3.0%	6	
20	Are you aware that there is a specialty that deals with the treatment of gum diseases?	Yes	71.0%	142	0.002*
		No	17.5%	35	
		Not sure	8.5%	17	
		Not responded	3.0%	6	

**Figure 1:**
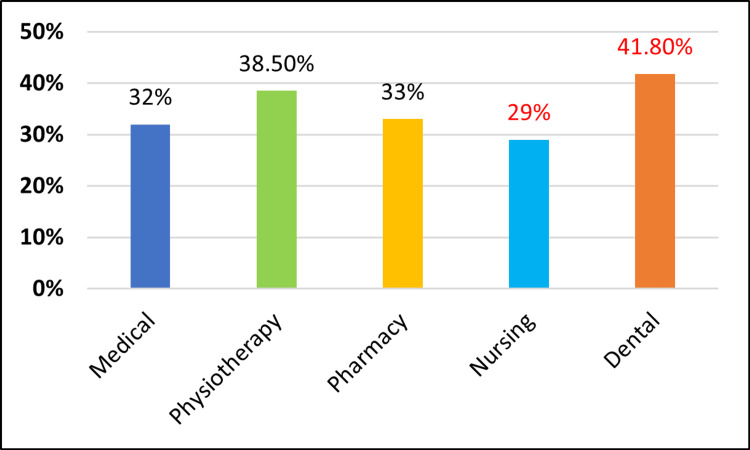
Comparison of knowledge regarding periodontal diseases among different faculties.

**Figure 2:**
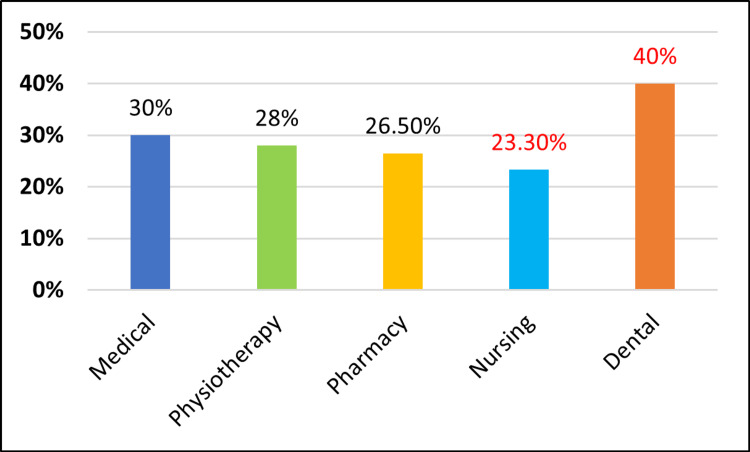
Comparison of attitudes regarding periodontal diseases among different faculties.

**Figure 3:**
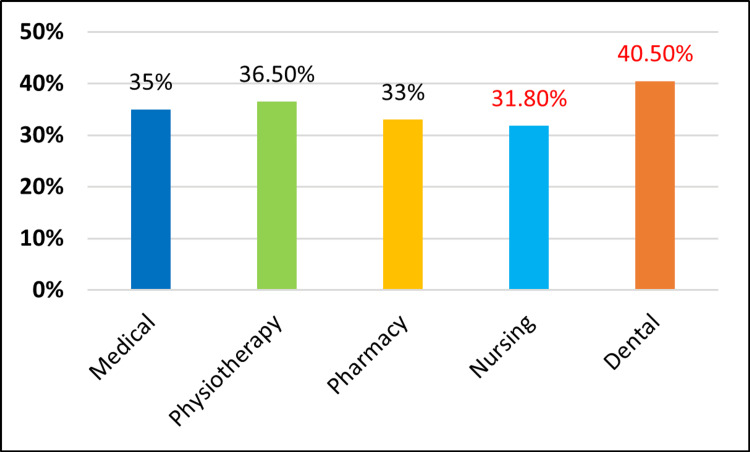
Comparison of practices regarding periodontal diseases among different faculties.

In our knowledge, attitudes, and practices (KAP) study, we found that first-year undergraduate students in the dental faculty have slightly better knowledge, followed by students in physiotherapy, medicine, pharmacy, and nursing (Figure 1). Dental students also showed a more positive attitude towards periodontal diseases, followed by students in medicine, physiotherapy, pharmacy, and nursing (Figure 2). Regarding oral hygiene practices, dental students have good oral hygiene practices, followed by students in physiotherapy, medicine, pharmacy, and nursing (Figure 3). The responses were better among dental students, which could be due to basic course information relating to oral health education, which might have had a positive impact.

## Discussion

The research conducted among first-year undergraduates from five professionals like medical, dental, physiotherapy, nursing, and pharmacy regarding their knowledge, attitude, and practices about periodontal diseases offers valuable insights into how aware and responsible students are towards oral and periodontal health. Scientific evidence has shown that periodontal diseases are complex diseases caused by the interaction between microorganisms and the host's immune and inflammatory responses [7]. Various systemic diseases and conditions can influence the progress of periodontitis or negatively impact the entire periodontium. Therefore, it is crucial to understand the connection between periodontal health and systemic diseases [8]. In 2016, Dayakar et al. surveyed to assess the level of awareness of periodontal health among 300 students from medical, ayurveda, and engineering colleges in the Kannada district. The survey revealed that even among health professionals and engineering students, there was a significant lack of understanding regarding oral hygiene and insufficient knowledge of oral hygiene practices [9]. In 2017, Penmetsa et al. conducted a study to assess the level of awareness of periodontal health and self-perception of halitosis among medical, pharmacy, and engineering students in the West Godavari district of Andhra Pradesh. The study revealed that although professional students had an acceptable level of knowledge regarding oral health, they lacked awareness when it came to periodontal health [10]. In 2017, Andhare surveyed 200 participants, including 100 medical and 100 dental undergraduate students. The survey concluded that medical students showed comparatively poor knowledge of oral health. It was noted that there was a lack of exposure to oral health in the medical curriculum. The study recommended that both medical and dental students should be encouraged to promote oral health education among their families, friends, and patients, aiming to be good role models for society [11]. In a 2019 cross-sectional study, Uma Sudhakar et al. found that there is an overall lack of knowledge about periodontal diseases among healthcare professionals. The study emphasizes the need for classes on periodontal diseases to better educate healthcare professionals and enable them to prevent these diseases and provide improved public health education [12]. In 2021, a descriptive cross-sectional survey was conducted by Banu et al. to evaluate the knowledge, attitude, and practices of undergraduate medical students in Madurai regarding oral health. The survey included 313 participants, and the results showed that 64.89% of final-year students, 65.21% of third-year students, and 60.61% of second-year students answered oral health-related questions correctly.

The study concluded that oral health awareness among undergraduate medical students was found to be satisfactory. However, the educational curriculum requires more extensive oral health programs to improve students' overall knowledge, attitudes, and practices [13]. In 2022, Gupta conducted a cross-sectional study of 560 practitioners of various medical fields in Kanpur city, concluding that dentists had a statistically higher level of awareness than physicians. Referral practice to periodontists was deficient by medical and alternate medical practitioners, despite the fact that they have good knowledge of the relationship between periodontal disease and systemic health [14]. Hence, interdisciplinary dental and medical training of practitioners is recommended. In 2022, Khan et al. conducted an online survey among 500 healthcare professionals in J&K to evaluate their awareness and motivation about periodontal health. The survey showed moderate awareness among respondents about periodontal health care. The study concluded that there is an urgent need for comprehensive educational programs to promote good oral health and impart education about correct oral hygiene practices [15]. Similarly, results from our study showed that the first-year undergraduate students from all five faculties lacked sufficient knowledge and demonstrated inadequate oral hygiene practices and attitudes toward oral and periodontal health and disease. Based on the results of our study, it is evident that healthcare professionals such as medical, dental, physiotherapy, pharmacy, and nursing professionals need to be made aware of proper oral hygiene practices. It is important to motivate these professionals to improve their behavior for the prevention and maintenance of periodontal disease. Furthermore, they should play a role in educating and motivating the general population about oral and periodontal diseases and referring patients who require treatment to a periodontist.

It is important to note that the study had certain limitations. Firstly, it only included first-year students and had a relatively small sample size. Additionally, the study was restricted to only one university. Therefore, caution must be exercised when attempting to generalize the results to university students in general. In the future, it would be beneficial to conduct similar studies with a larger sample size in order to obtain more accurate and representative results.

## Conclusions

Based on the observations of our study, it is evident that the knowledge, attitude, and behavior related to periodontal disease among first-year undergraduates need improvement in a few areas. Dental students had better knowledge, attitudes, and practices regarding periodontal diseases compared to students from other faculties. Conversely, nursing students exhibited the least knowledge, attitude, and practices related to periodontal diseases. Healthcare professionals serve as role models for the public and are required to have good periodontal health attitudes and behaviors. Allied and paramedical students should be taken on board to improve their basic knowledge regarding preventive oral care. Therefore, it is important to incorporate oral and periodontal disease knowledge into the curriculum not only for healthcare and allied professionals but also for secondary school students. This educational initiative would help healthcare providers to enhance their knowledge regarding oral health. Improved knowledge among medical and allied practitioners regarding periodontal health will ultimately benefit the population at large by spreading awareness of the need of healthy oral practices.
